# Mitigating SARS-CoV-2 Transmission in Hospitals: A Systematic Literature Review

**DOI:** 10.3389/phrs.2022.1604572

**Published:** 2022-02-23

**Authors:** Chester Yan Hao Ng, Nicole-Ann Lim, Lena X. Y. Bao, Amy M. L. Quek, Raymond C. S. Seet

**Affiliations:** ^1^ Department of Medicine, Yong Loo Lin School of Medicine, National University of Singapore, Singapore, Singapore; ^2^ Healthy Longevity Translational Research Program, Yong Loo Lin School of Medicine, National University of Singapore, Singapore, Singapore

**Keywords:** COVID-19, SARS-CoV-2, hospital outbreaks, nosocomial spread, infection control

## Abstract

**Objectives:** Hospital outbreaks of SARS-CoV-2 infection are dreaded but preventable catastrophes. We review the literature to examine the pattern of SARS-CoV-2 transmission in hospitals and identify potential vulnerabilities to mitigate the risk of infection.

**Methods:** Three electronic databases (PubMed, Embase and Scopus) were searched from inception to July 27, 2021 for publications reporting SARS-CoV-2 outbreaks in hospital. Relevant articles and grey literature reports were hand-searched.

**Results:** Twenty-seven articles that described 35 SARS-CoV-2 outbreaks were included. Despite epidemiological investigations, the primary case could not be identified in 37% of outbreaks. Healthcare workers accounted for 40% of primary cases (doctors 17%, followed by ancillary staff 11%). Mortality among infected patients was approximately 15%. By contrast, none of the infected HCWs died. Several concerning patterns were identified, including infections involving ancillary staff and healthcare worker infections from the community and household contacts.

**Conclusion:** Continuous efforts to train-retrain and enforce correct personal protective equipment use and regular routine screening tests (especially among ancillary staff) are necessary to stem future hospital outbreaks of SARS-CoV-2.

## Introduction

The SARS-CoV-2 virus has inflicted a global health crisis, infecting at least 271 million individuals and causing over 5.3 million deaths as of December 18, 2021 [1]. Compared with other coronaviruses (mainly SARS-CoV and MERS-CoV), the SARS-CoV-2 virus is highly transmissible especially in unvaccinated and immunologically naïve populations [2, 3]. Despite causing a largely asymptomatic and mild disease, COVID-19 infection could lead to multi-organ damage, pneumonia and death, especially among unvaccinated elderly individuals with multiple comorbidities [4]. Early during this pandemic, uncertainties surrounding the mode of transmission and scarcity in personal protective equipment (PPE) supplies [5] contributed to high rates of infections in countries worldwide. Healthcare systems were continuously strained by the need to care for more severe COVID-19 patients, whilst ensuring the medical needs for existing patients with other acute and chronic medical illnesses are not compromised [6]. A large prospective cohort study of over 2 million individuals in the United States and United Kingdom observed a 12-fold increase in risk of SARS-CoV-2 infection involving frontline healthcare workers (HCWs) compared to community individuals, with inpatient HCWs in particular facing an even greater 24-fold increase in risk [7]. Frontline HCWs with direct contact with patients (e.g., doctors, nurses and allied health personnel) were subjected to significant work-related fatigue from caring for their patients for prolonged hours and for continuously being in a state of heightened alert [8]. Countries with more developed healthcare systems (e.g., the United States, Italy and Spain) also witnessed high infection rates. In the first few months of the pandemic, a sero-surveillance study involving over 6,000 Spanish HCWs identified 662 individuals (11.0%) with SARS-CoV-2 seropositivity [9]. In Taiwan and Singapore where enforcement of nationwide restrictions initially kept infections at a very low level, a resurgence in cases occurred among their HCWs close to a year after SARS-CoV-2 infection was first detected [10, 11]. Despite high vaccination rates, a resurgence of SARS-CoV-2 infection was similarly observed among healthcare workers at the University of California San Diego Health [12], coinciding with the emergence of the more transmissible variants of concern [13].

The first silver lining of the pandemic occurred when no infections were reported among 42,322 returning medical staff deployed from various parts of China to assist in the Wuhan outbreak, testifying to the overall efficacy of non-pharmacological interventions (NPI) to prevent nosocomial transmission [14]. NPIs refer to measures aimed at disrupting transmission risks through early isolation and quarantine, physical distancing, use of face masks and hand hygiene [15]. In hospitals, NPI measures mandate the use of PPE (comprising N95 respirators, eye protection, full sleeve gown, and gloves) among frontline HCWs when attending to suspected or confirmed COVID-19 patients and among laboratory staff handling biological samples from these patients. In other clinical settings, PPE use is tiered according to work area, patient-care activity, or procedure performed, prioritizing frontline HCWs such as doctors and nurses [15]. However, despite vaccination and NPI measures [16], outbreaks of SARS-CoV-2 continue to be reported in various healthcare settings. This review aims to systematically examine the pattern of SARS-CoV-2 outbreaks in hospitals and identify vulnerable aspects of our preparedness against future outbreaks.

## Methods

The Preferred Reporting Items of Systematic Reviews and Meta-Analyses (PRISMA) reporting guideline was used in the synthesis of this review [17]. A literature search of 3 electronic databases (PubMed, Embase and Scopus) was conducted from inception to July 27, 2021. The following keywords were used in combination: (SARS-CoV-2 OR COVID-19 OR 2019‐nCoV OR 2019 AND ncov) AND (hospital OR healthcare worker) AND outbreak. The appropriate Medical Subject Heading (MeSH) terms and Emtree terms were used in PubMed and Embase respectively. The exact search strategy is presented in Supplementary Table S1. Only articles written in English were included. In addition, references cited in relevant studies were hand searched and grey literature reports from official sources were identified *via* web searches.

The articles retrieved from the search were independently screened by two authors and relevant data were extracted, with disagreements resolved by a third senior author. We included any publication types, including full text articles and abstracts, that reported SARS-CoV-2 outbreaks or clusters in hospitals, which were defined as two or more linked cases of COVID-19 within a hospital setting. Data extracted included primary case, detection method and origin of transmission, country of origin, date of first confirmed case, days until no new cases, number of HCWs and patients infected and the respective attack and mortality rates. Key features and learning points of each outbreak were also retrieved. Statistical analysis was performed using R version 4.0.2 [18]. Proportions are presented in percentages and continuous variables are presented as mean with standard deviation. A PRISMA checklist is included in Supplementary Figure S1.

## Results

**FIGURE 1:**
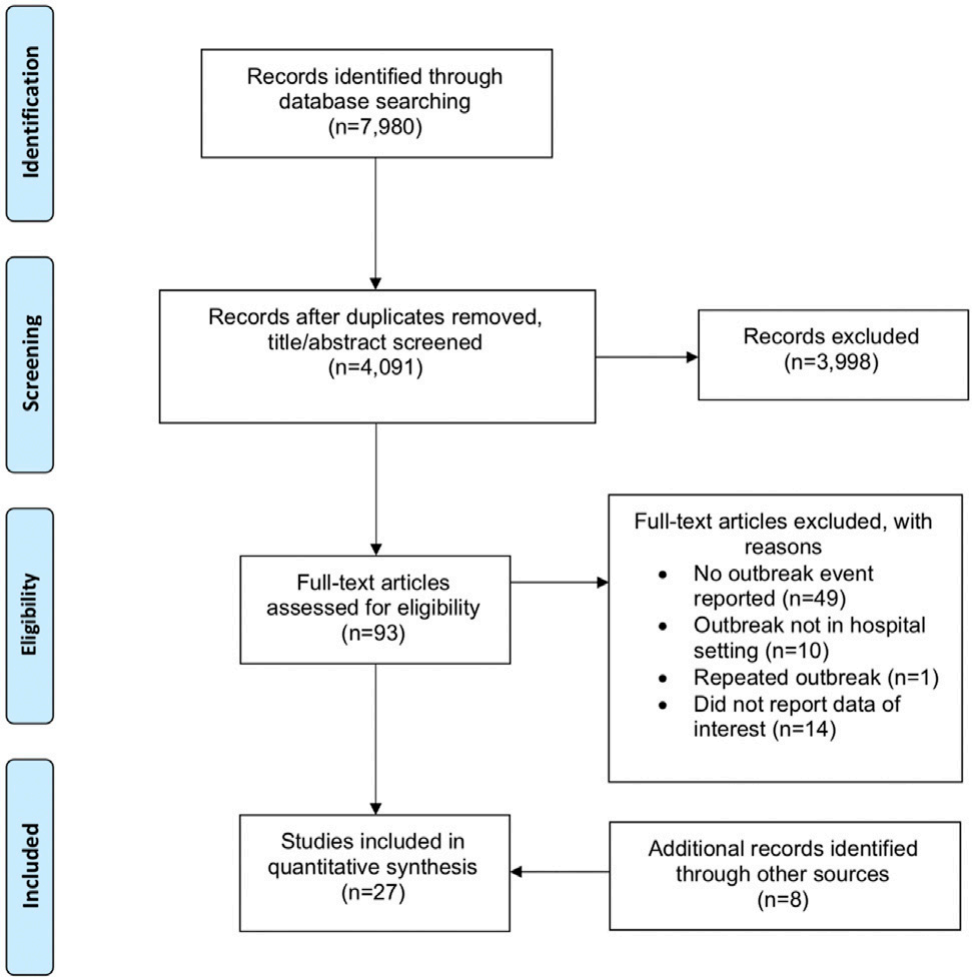
Preferred reporting items of Systematic Reviews and Meta-Analyses diagram of study selection (Singapore, 2021).

A total of 7,980 articles were identified from the initial database search, which was reduced to 4,091 after duplicate publications were removed. Based on the title and abstract, 3,998 articles were excluded. Full text screening further excluded 74 studies. Five additional articles were hand searched from citations and 3 grey literature articles were identified from web searches. Overall, 27 articles reporting 35 outbreaks or clusters were included [10, 11, 19–43]. A flow diagram of the study selection is shown in Figure 1.

### Outbreak Characteristics

Thirty-five SARS-CoV-2 outbreaks occurring in 32 hospitals were included. Seven occurred in Germany, 6 in South Korea, 3 each in the United States, China and Vietnam, 2 each in Singapore, the United Kingdom and France, and 1 each from Japan, South Africa, Poland, Portugal, Nepal, Belgium and Taiwan. The outbreaks occurred between January 2020 and June 2021. The mean average time from the first positive individual in the outbreak to no new cases was 18.4 (±13.6) days.

Earlier outbreaks were characterized by a lack of awareness or implementation of NPI measures [19, 20, 30]. In outbreaks where NPI measures were implemented and enforced, asymptomatic spread as well as missed or delayed diagnosis of primary cases originating from community transmission led to uncontrolled nosocomial spread [22, 25]. Outbreaks in Germany and South Korea later in the pandemic identified HCW households in particular as likely sources of infection [35, 41]. Selhorst et al. reported reinfection in a HCW despite significant neutralizing antibodies [40] while outbreaks in Singapore and Taiwan resulted from transmission of Delta and Epsilon variant strains among HCWs respectively [10, 11]. Four outbreaks, occurring in Singapore and Vietnam, involved HCWs who were vaccinated [10, 43]. Eleven (31.4%) outbreaks included in the review highlighted lapses in adherence to NPI measures as a potential reason for the outbreak [19, 20, 24, 30–35, 39, 41]. However, the use of genomic sequencing to establish links between SARS-CoV-2 cases was only described in 4 outbreaks [32, 37, 40, 42]. Detailed information and key features of each outbreak are summarized in Table 1.

**TABLE 1:** Characteristics of SARS-CoV-2 outbreaks in hospitals (Singapore, 2021).

Number	Author	Country	First confirmed case	Primary case	Detection method	Origin of transmission	Days until no new cases	Key features
1	Gao et al [19]	China	January 2020	Unclear, 2 patients	Symptomatic testing	No prior exposure	Not reported	Lack of awareness of NPI during the early stages of the pandemic
								Atypical presentations of COVID-19 patients
2	Ji et al. [20]	China	January 2020	Unclear	Symptomatic testing	No prior exposure	Not reported	Patients with mental illness could not communicate symptoms
3		South Korea	February 2020	Unclear, likely patient	Symptomatic testing	No prior exposure	Not reported	Psychiatric ward densely packed with lack of ventilation and hand sanitizer
4	Kim et al. [21]	South Korea	February 2020	HCW, patient transfer staff	Symptomatic testing	No prior exposure	5	Largely minimally symptomatic or asymptomatic spread
5	Asad et al. [22]	United Kingdom	Early 2020	Unclear, likely asymptomatic HCW	Symptomatic testing	No prior exposure	14	Hand hygiene compliance was 100% within ward but lack of safe distancing within ward areas
								Largely minimally symptomatic or asymptomatic spread
6	Schwierzeck et al. [23]	Germany	Early 2020	HCW, role of HCW was not provided	Symptomatic testing	No prior exposure	4	Corroborative findings that “better positive predictive values if tests are performed in symptomatic individuals”
7	Baker et al. [24]	United States	Early 2020	Patient	Symptomatic testing	No prior exposure	Not reported	Atypical presentation with delayed diagnosis of primary case
8	Harada et al. [25]	Japan	March 2020	Patient	Asymptomatic surveillance	Exposure from previous hospital	Not reported	Universal masking and employee health screening measures in place
								Largely minimally symptomatic or asymptomatic spread
9	Vanhems et al. [26]	France	March 2020	Unclear, 2 patients	Symptomatic testing	No prior exposure	8	Rapid spread of infection
10	Luong-Nguyen et al. [27]	France	March 2020	Unclear	Symptomatic testing	No prior exposure	Not reported	Systemic screening limited by sensitivity of PCR testing and viral incubation time
								Reduce hospital stay by utilizing outpatient services
11	Rickman et al. [28]	United Kingdom	March 2020	Unclear	Symptomatic testing	No prior exposure	Not reported	In 32% of infections, no source was identified, likely from asymptomatic or undiagnosed patients, visitors or HCWs
12	Jung et al. [29]	South Korea	March 2020	Patient	Symptomatic testing	Previous contact with COVID-19 patient	23	Transmission from asymptomatic children to adults
								Successful containment with extensive contact tracing and testing
13	Brandt et al. [30]	Germany	March 2020	HCW, role of HCW was not provided	Symptomatic testing	No prior exposure	25	Four possible primary cases who acquired infection outside of the hospital
								Lack of HCW mask wearing until outbreak
14	Schneider et al. [31]	Germany	March 2020	Outbreak 1: HCW, doctor	Symptomatic testing	No prior exposure	7	HCW to HCW transmission may be a critical factor in the spread of COVID-19 outbreaks in hospitals and could outweigh the risks posed by infected patients
15				Outbreak 2: HCW, nurse	Asymptomatic surveillance	No prior exposure	18	
16				Outbreak 3: HCW, doctor	Symptomatic testing	No prior exposure	12	
17				Outbreak 4: HCW, doctor	Symptomatic testing	No prior exposure	17	
18	Lessells et al. [32]	South Africa	March 2020	Unclear, likely patient	Symptomatic testing	No prior exposure	53	Atypical presentations of COVID-19 patients
								Lack of early intervention
19	Duy et al. [33]	Vietnam	March 2020	Unclear	Symptomatic testing	No prior exposure	Not reported	Inadequate compliance to infection control measures by nonmedical staff
								Whole hospital quarantine for more than 2 weeks was implemented
								Largely minimally symptomatic or asymptomatic spread
20	Hale et al. [34]	United States	March 2020	HCW, room service ambassador	Symptomatic testing	No prior exposure, 2 have symptomatic family members, 1 deployed to another healthcare facility	19	Staff had common locker room and break area
								Food service worker worked in another healthcare facility
21	Höring et al. [35]	Germany	April 2020	Unclear, 1 HCW and 1 patient	Symptomatic testing and admission testing	No prior exposure	16	Group of religious HCWs lived in same household and attended service together
								Largely minimally symptomatic or asymptomatic spread
22	Biernat et al. [36]	Poland	April 2020	HCW, nurse	Symptomatic testing	No prior exposure	Not reported	High mortality rate among hematological patients with COVID-19
								Asymptomatic surveillance every 7 days for HCWs
23	Borges et al. [37]	Portugal	August 2020	Unclear	Admission testing	No prior exposure	13	Largely minimally symptomatic or asymptomatic spread
								The combination of epidemiological and genomic data is beneficial when investigating complex outbreaks
24	Dhakal et al. [38]	Nepal	August 2020	HCW, nonclinical staff	Symptomatic testing	No prior exposure	59	Outbreak despite strict masking, hand hygiene and education
								Continued expansion of telerehabilitation capacity
25	Klompas et al. [39]	United States	September 2020	Patient	Symptomatic testing	No prior exposure	20	Surgical masks can reduce but not fully eliminate aerosol and viral exposure
								Multiple sets of infected roommates even without infected staff intermediary
26	Selhorst et al. [40]	Belgium	September 2020	Patient	Symptomatic testing	No prior exposure	5	Reinfection in HCW after primary infection despite significant neutralizing antibodies
27	Lee et al. [41]	South Korea	November 2020	HCW, doctor	Symptomatic testing	Community contact	Not reported	Initial source of infection was from the community
28				Caregiver	Screening after outbreak	No prior exposure	Not reported	Largely minimally symptomatic or asymptomatic spread
29				HCW, doctor	Screening after outbreak	Wife’s family tested positive	Not reported	Hand hygiene and mask wearing compliance variable
30	Cheng et al. [42]	Hong Kong, China	December 2020	Patient	Symptomatic testing	No prior exposure	9	Primary case not picked up on admission testing
								Likely airborne transmission with no air exhaust grilles inside semi-enclosed patient cubicles
31	Akhmetzhanova et al. [11]	Taiwan	January 2021	Patient	Symptomatic testing	Positive travel history	26	Outbreak despite strict measures including mask wearing
								Epsilon variant detected in subset of cases
32	Ministry of Health, Singapore [10]	Singapore	April 2021	Unclear, likely HCW, nurse	Symptomatic testing	No prior exposure	13	Outbreak originated from Delta variant breakthrough infection in vaccinated HCWs, including cleaners and porters
33		Singapore	June 2021	HCW, porter	Asymptomatic surveillance	No prior exposure	12	Large scale ringfencing and quarantining of contacts, lockdown of affected wards and restriction on new admissions
34	World Health Organization [43]	Vietnam	May 2021	HCW, doctor	Travel testing	No prior exposure	Not reported	Breakthrough infections among vaccinated HCWs, including cleaners
35		Vietnam	May 2021	Patient	Asymptomatic surveillance	Exposure from previous hospital	26	Significant spread of infection to patients

COVID-19, coronavirus disease 2019; HCW, healthcare worker; NPI, non-pharmacological interventions; PCR, polymerase chain reaction.

### Primary Case

**FIGURE 2:**
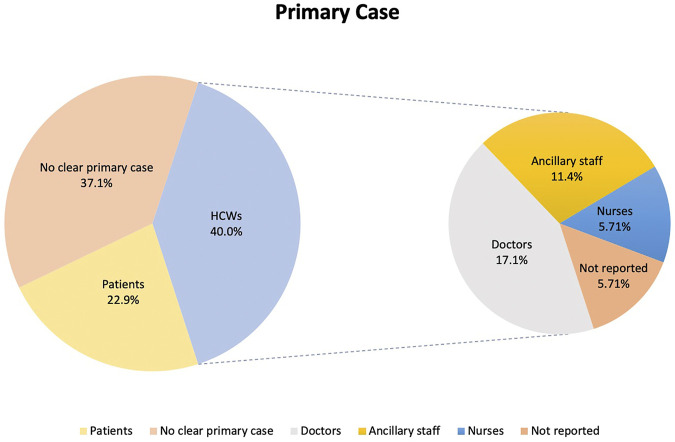
Distribution of primary cases according to healthcare workers, patients and others (Singapore, 2021).

Primary cases are defined in this study as the first infected person to bring SARS-CoV-2 infection into the hospital setting [44]. In 14 (40.0%) of the outbreaks the primary case was a HCW, of whom 6 (17.1%) were doctors, 4 (11.4%) were ancillary staff (either patient transfer staff, room service ambassador, nonclinical staff, or porter), 2 (5.71%) were nurses. The roles of the remaining 2 (5.71%) HCW primary cases were not provided in the primary publications. Eight (22.9%) of the outbreaks were attributed to a patient primary case and in 13 (37.1%) outbreaks no clear primary case was reported. Twenty-seven (77.1%) of the outbreaks were detected from symptomatic testing, 4 (11.4%) from asymptomatic surveillance, 2 (5.71%) from screening after an outbreak and 1 (2.86%) each from admission testing and travel testing. Only 6 (17.1%) of the primary cases had reported prior contact with any COVID-19 positive individuals. The data is summarized in Figure 2.

### Infection Demographics

**FIGURE 3:**
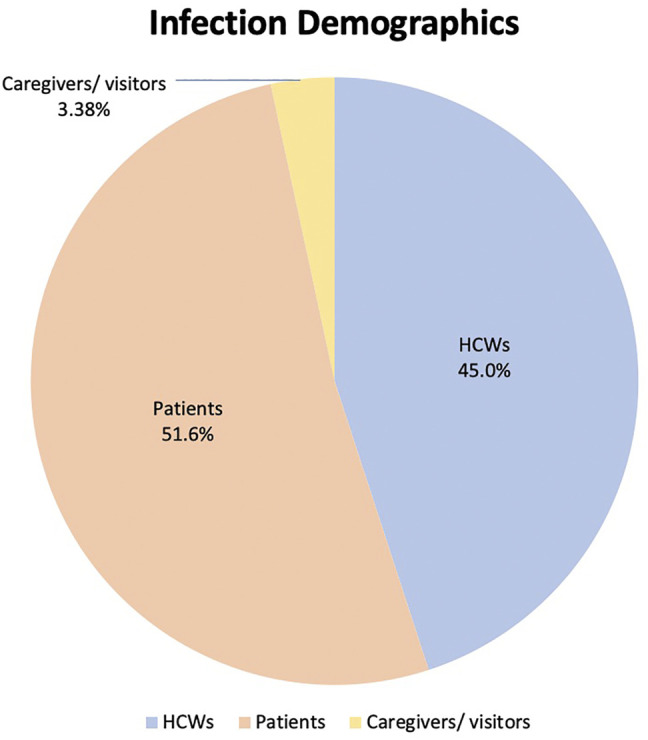
Distribution of infected patients and healthcare workers (Singapore, 2021).

Thirty-two outbreaks reported breakdowns of SARS-CoV-2 infection demographics, comprising 1,007 infected individuals. HCWs accounted for 453 (45.0%) of total infected individuals, patients accounted for 520 (51.6%) and the remaining 34 (3.38%) comprised either caregivers or visitors, as presented in Figure 3.

### Attack and Mortality Rates

Six outbreaks reported attack rate among HCWs (21.7 ± 17.4%), and 6 outbreaks reported attack rate among patients (21.4 ± 16.3%). No HCWs died across 29 outbreaks reporting HCW mortality, while 28 outbreaks reported on patient mortality rate (15.0 ± 20.7%), summarized in Supplementary Table S2. Mortality rate was highest in an outbreak involving patients with underlying hematological disorders (50.0%).

## Discussion

**FIGURE 4:**
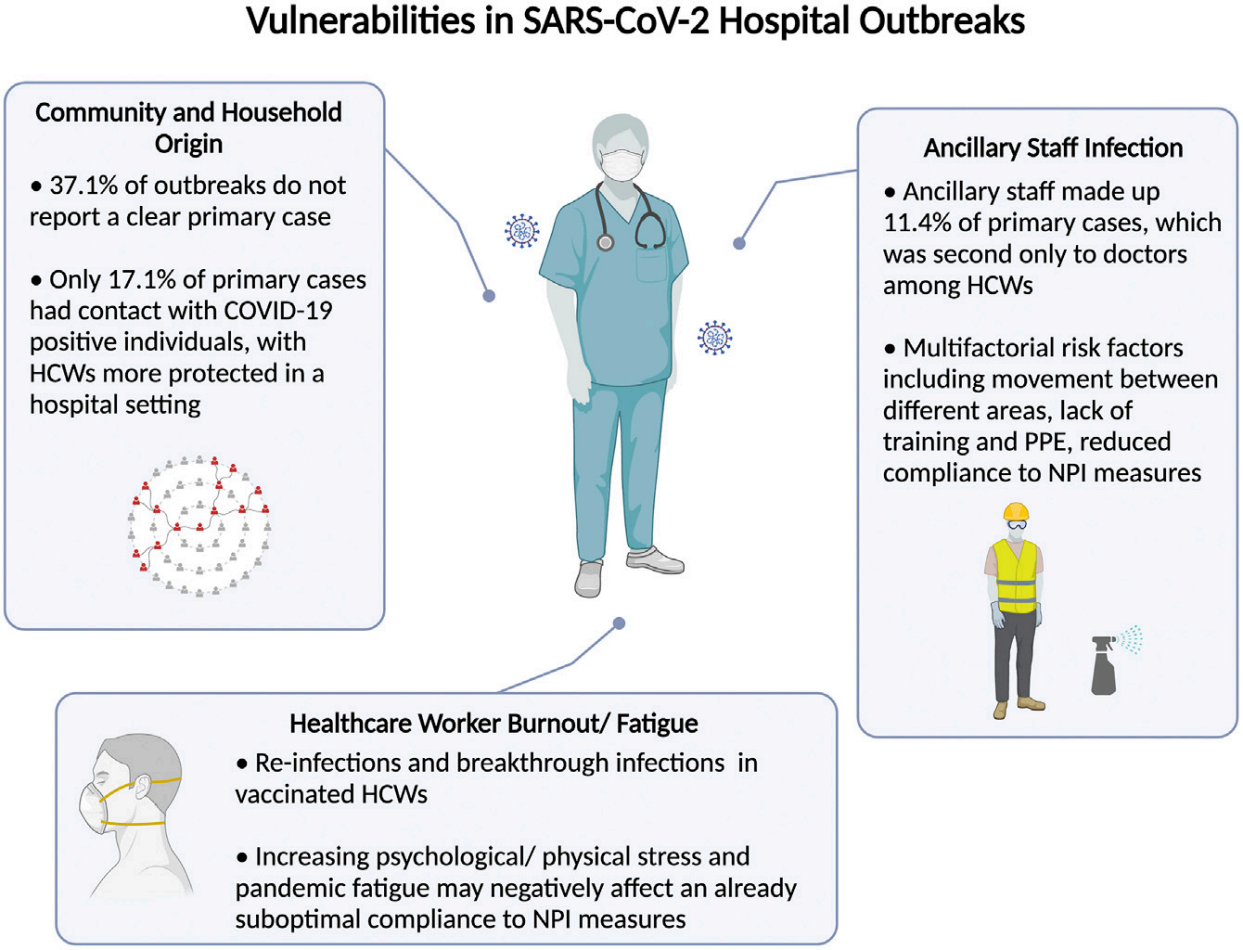
Summary of identified vulnerabilities control leading to severe acute respiratory syndrome coronavirus 2 hospital outbreaks (Singapore, 2021).

The current COVID-19 pandemic continues to strain healthcare systems worldwide. Despite better access to personal protective equipment and stringent hospital NPI measures, the risk of transmission in hospitals continues to beleaguer healthcare leaders and policymakers worldwide. This systematic review characterizes the pattern of hospital transmission and identifies important vulnerabilities in existing hospital preparedness against future infections. Data from this review highlight ancillary staff as primary cases of reported outbreaks, reverse transmission from the community and household contacts, and a resurgence of infections among HCWs despite vaccination and NPI measures (Figure 4).

A key observation from the current study is a significant predisposition of non-clinical ancillary staff working as cleaners, porters, housekeeping staff and healthcare assistants to developing SARS-CoV-2 infection. In the current review, ancillary staff formed the second largest group of HCW primary cases, second only to doctors. Although doctors, nurses and other frontline HCWs are closely scrutinized for PPE and NPI compliance [45], the critical roles played by ancillary staff in disrupting the chain of infection have received less attention. In contrast to the work environment of doctors and nurses that are regularly cleaned and decontaminated, the work areas of ancillary staff could vary substantially depending on their vocations. Some ancillary staff are involved in the transport of suspected or confirmed COVID-19 patients, clean rooms of patients whose infection status were previously unknown, and share a communal area to rest and dine. Although ancillary staff made up of approximately 10% of primary HCW cases in the current study, these figures are also likely an underestimate as many articles do not specify the type of HCWs and consider occupational roles as either doctors, nurses and “other” healthcare staff. An increasing number of epidemiological studies have also reported not only a greater risk of infection but also highlighted ancillary staff as the main affected group within their cohorts [46–51] (Table 2). Several factors could explain increased propensity of ancillary staff to infection. For example, porters move between different spaces within hospital settings, including hospital wards, radiology diagnostic centers, emergency rooms and clinics, and have numerous contacts with patients and other HCWs. As services requiring ancillary staff are often contracted to external vendors, it is also not uncommon for cleaners and catering staff to rotate through different environments and buildings, as reported in an outbreak involving a food service worker in an American hospital who worked in multiple settings [34]. Another report in Vietnam identified non-compliance to NPI measures among nonmedical staff contributing to an outbreak involving 27 catering staff, where a lack of training, personal protective supplies, and underestimation of virus transmission were cited as possible explanations [33]. Due to the more physical nature of their job, ancillary staff are also less likely to fully comply to PPE and are thus less protected. They may not be subject to adequate NPI training and the same scrutiny imposed on medical HCWs. Housekeeping staff and hospital cleaners too harbor increased risk of infection, as they are more likely to encounter contaminated surfaces [52]. One study reported lower rates of compliance to hand hygiene among non-medical HCWs [53].

**TABLE 2:** Epidemiological studies reporting ancillary staff as main group of COVID-19 healthcare worker infections (Singapore, 2021).

Author	Year	Country	Design	Cohort size	Outcome
Eyre et al. [46]	2020	United Kingdom	Cross-sectional	10,034	Porters and cleaners had the highest rates of infection (18.6%) out of all occupational roles
Shields et al. [47]	2020	United Kingdom	Cross-sectional	545	Housekeeping staff were the largest group of seropositive HCWs (34.5%), compared to 14.8% among those working in intensive care medicine
Alkurt et al. [48]	2021	Turkey	Cross-sectional	932	Cleaning staff seropositivity rates (6%) were highest among HCWs in 3 hospitals
Wong et al. [49]	2021	Singapore	Cross-sectional	5,050	Ancillary staff made up the biggest group (48.6%) out of all locally acquired cases in Singapore between January, 23 and April 17, 2020
Al-Kuwari et al. [50]	2021	Qatar	Cross-sectional	1,048	Storekeepers, engineering and maintenance staff, housekeeping staff, support staff, and security staff had the highest infection rates of 100, 67.2, 47.1, 32.4 and 29.5% respectively
Dev et al. [51]	2021	India	Case-control	3,100	Sanitation workers were at highest risk of infection [RR 3.24 (95% CI 2.05–5.12), *p*-value <0.0001]

COVID-19, coronavirus disease 2019; HCW, healthcare worker.

With better understanding of the mechanics of viral transmission, hospitals have grown to be safer workplaces, compared to earlier in the pandemic where frontline HCWs were at significantly increased risk of infection [7]. Infection rates involving doctors and nurses have fallen substantially compared with those at the start of the pandemic. When the second wave of infections hit the United States in mid-2020, Baker et al. reported that out of a cohort of 10,275 HCWs, those who reported caring for patients with COVID-19 did not have increased risk of seropositivity [adjusted OR (95% CI) = 0.9 (0.7–1.3)] [54], suggesting that the risk of transmission from their daily work is low as a result of improved NPI measures. Data from this review, however, identify potential weak links for hospital outbreaks to be unlinked cases and the transmission of infection *via* asymptomatic primary HCWs not originating within the hospital. Close to 40% of hospital outbreaks did not report a clear primary case and most primary cases (82.9%) did not have prior contact with COVID-19 positive patients. An earlier study in 2 Dutch hospitals also found 24% of infected HCWs had no patient contact at all and only 3.4% were exposed to a known inpatient with COVID-19 [55]. This is supported by results from viral sequencing among United States HCWs from 25 March to December 27, 2020 revealing that majority of infections were not linked to patients or co-workers and were instead genetically similar to viruses that were circulating in the community [56]. Among community settings, households in particular are higher risk settings where prolonged contact between household members could increase the likelihood of transmission. Furthermore, SARS-CoV-2 transmission is more likely in an indoor setting where the estimated household attack rate is 16.6%, significantly higher than that of previous coronavirus outbreaks [57]. The risk of bringing SARS-CoV-2 infection from the household to the hospital was highlighted by hospital outbreaks in Germany and South Korea [35, 41]. Ran et al. reported early in the pandemic that HCWs in Wuhan with a diagnosed family member had a significantly increased risk of infection [RR (95% CI) = 2.76 (95% CI 2.02–3.77)] [58]. The risk of reverse transmission from the community and households could be compounded further by measures to self-isolate together with household members who share a common living environment and facilities (e.g., bathrooms) and in settings with uncontrolled community transmission. Central to reducing hospital outbreaks is also the control of community rates of transmission.

Despite compelling data supporting the overall efficacy of vaccination, breakthrough infections are known to occur due to various factors, including suboptimal production of antibodies, waning immunity and emergence of variant strains [59]. In Singapore and Taiwan, the emergence of more transmissible strains of the virus (e.g., Delta and Epsilon variant strains) had led to nosocomial spread of the SARS-CoV-2 virus even among vaccinated HCWs [10, 11]. The rise in breakthrough infections may also be compounded by increasing pandemic fatigue among HCWs. A cross-sectional survey of 5 major cities observed decreased mask wearing and pandemic mitigation measures when declining incidence led to a lower perceived severity of COVID-19 [60]. Furthermore, as the pandemic progressed, HCWs have struggled with increased workload, longer working hours, prolonged states of heightened alert and restrictions in social mobility, with reports of increased depression, anxiety and stress stemming from fear and stress at the workplace as well as added stigma from the general public [61]. Apart from psychological implications, HCWs also face physical challenges from extended PPE usage. A systematic review of 14 studies estimated overall prevalence of PPE related adverse events to be 78% (95% CI 66.7–87.5%), including headache, dry skin and dyspnea [62]. Although poor wellbeing is commonly associated with medical errors and poorer patient safety outcomes [63], few studies have examined the possible link between burnout and compliance to NPI measures. Earlier in the pandemic, Zhou et al. found that increased burnout was negatively associated with hand hygiene in a study of 1,734 HCWs from 17 medical teams in Wuhan [64], corroborating pre-pandemic findings in a Greek hospital [65]. Despite playing a critical role, the pandemic has taken a toll on the mental wellbeing of HCWs which could compromise their adherence with NPI measures from work-related fatigue and burnout, compounding the already suboptimal compliance which led to 31.4% of the outbreaks in this review. This is especially important as HCWs often share common areas such as toilets, pantries and locker rooms [34]. These areas where NPI measures are likely to be less stringent could account for outbreaks where measures were supposedly well followed, leading to HCWs comprising 45.0% of infected individuals.

To implement NPI measures during severe outbreaks, HCWs should be allowed to isolate themselves from their families and the community, in temporary accommodation facilities that allow HCW to exercise physical distancing. Hotel facilities with staff cooperation and proper planning have been demonstrated to be viable options to house HCWs without the risk of SARS-CoV-2 spread [66]. Wang et al. also reported the effective use of face masks and disinfectants within households to reduce spread between family members [67], which can be applied when full isolation from potential household contacts is not possible. In these circumstances, pharmacological interventions such as povidone-iodine throat spray could be employed to reduce transmission risks to supplement NPI measures. In a large-scale study involving residents living in a workers’ dormitory, povidone-iodine throat spray was found to be associated with a 24% absolute risk reduction compared with an active comparator group who received oral vitamin C [68]. Measures to protect the safety of ancillary staff could define whether transmission involving HCWs could hinder the remarkable progress to lower HCW infection. Ancillary staff should be given the same intensity of NPI training as medical staff where personal protective supplies are adequate and their compliance to NPI measures is enforced. Engagement with healthcare workers to understand their views on policy and addressing practical issues such poor visibility from eye protection would aid in improving compliance [69]. Periodic surveillance using antigen rapid tests (ART) or polymerase chain reaction (PCR) tests could be implemented to facilitate early isolation. Although routine surveillance testing has not gained wide acceptance, these surveillance tests have identified asymptomatic cases that would have otherwise been missed, not just for HCWs but inpatients as well. Twice-weekly routine testing has been recommended in the setting of ongoing community transmission, though it has to be supplemented by NPI measures [70]. Studies have demonstrated the viability of maintaining high levels of compliance to regular screening in universities, where there is similarly shared accommodation and abundant interactions [71]. Retrospective investigation and open reporting of hospital outbreaks is also crucial, with 37.1% of outbreaks included in this review not reporting a clear primary case. The availability of additional data will allow for future studies to understand the pattern of infection chains and identify vulnerabilities in existing protocols. Vaccination remains imperative despite the emergence of new SARS-CoV-2 strains, by substantially reducing viral loads in infected individuals and mitigating infectiousness [72]. Emerging data from Israel also reflect lower rates of COVID-19 and severe illness among the elderly after the administration of a booster vaccine dose [73].

Several limitations merit mention. First and most significantly, due to our search endpoint of July 27, 2021 the outbreaks included mostly included unvaccinated individuals, with many countries still working on vaccinating their populations. Since then, vaccination has been rolled out at different rates worldwide, with some countries offering different vaccine options. Although it is possible vaccination could alter transmission dynamics of the SARS-CoV-2 virus, the lessons gleaned from this review are likely to remain relevant and applicable to most healthcare settings especially with the emergence of more transmissible variants of concern, and the different availability and rates of booster vaccination among HCWs worldwide. Second, we relied on data from the published literature and did not systematically approach individual centers for the incidence of COVID-19 infection among their HCWs. This may lead to publication bias as attempts to report research findings towards the later part of the pandemic may not be favorably reviewed and accepted for publication. Third, there was significant heterogeneity when reporting HCW infections in ancillary staff which may understate the burden of infection in this segment of HCWs. Fourth, we only considered outbreaks in the hospital setting and did not examine primary care clinics or long-term care facilities such as nursing homes. Fifth, articles included in this review originated from high-income countries with adequate access to PPE supplies and good hospital infrastructure, and may not be generalizable to poor-resource healthcare settings. Sixth, only 4 of the papers reporting outbreaks described the use of genomic sequencing in establishing infection links, whereas ascertainment of primary cases for the remaining studies was based on traditional contact tracing which may not be definitive.

This systematic review highlights important learning points that elucidated on factors leading to outbreaks of SARS-CoV-2 infection in healthcare settings. Although there is a gradual shift towards considering COVID-19 as an endemic disease and economic considerations to loosen existing restrictions, active measures are needed to ensure patients can recover in a safe hospital environment without fear of contracting the virus. Caution should be exercised to prevent complacency with PPE measures to result in future hospital outbreaks as this could have devastating consequences to patients and the morale of HCWs [33]. Continuous efforts to train-retrain PPE measures especially among ancillary staff, encourage vaccination and implement regular routine screening will aid in detecting and stemming transmission of the virus early. The pandemic has exposed important vulnerabilities within healthcare systems which should be addressed before they are further exploited by the virus.
